# Initial Assessment of the Acceptability of a Push-Pull *Aedes aegypti* Control Strategy in Iquitos, Peru and Kanchanaburi, Thailand

**DOI:** 10.4269/ajtmh.2011.09-0615

**Published:** 2011-02-04

**Authors:** Valerie A. Paz-Soldan, Valaikanya Plasai, Amy C. Morrison, Esther J. Rios-Lopez, Shirly Guedez-Gonzales, John P. Grieco, Kirk Mundal, Theeraphap Chareonviriyaphap, Nicole L. Achee

**Affiliations:** International Health and Development Department, Tulane University School of Public Health and Tropical Medicine, New Orleans, Louisiana; Bureau of the Vector-borne Diseases, Department of Disease Control, Ministry of Public Health, Nonthaburi, Thailand; Department of Entomology, University of California, Davis, California; United States Naval Medical Research Center Detachment, Iquitos, Peru; Department of Preventive Medicine and Biometrics, Uniformed Services University of the Health Sciences, Bethesda, Maryland; Department of Entomology, United States Naval Medical Research Center Detachment, Lima, Peru; Department of Entomology, Faculty of Agriculture, Kasetsart University, Bangkok, Thailand

## Abstract

As part of a larger research program evaluating chemical threshold levels for a Push-Pull intervention to reduce man-vector (*Aedes aegypti*) contact, this qualitative study explored local perceptions and strategies associated with mosquito control within dengue-endemic communities in Peru and Thailand. Focus groups were used to provide preliminary information that would identify possible public acceptance issues to the Push-Pull strategy in each site. Nine focus group discussions (total of 102 individuals) conducted between September 2008 and March 2009 examined several themes: 1) current mosquito control practices; 2) perceptions of spatial repellency and contact irritancy versus killing mosquitoes; and 3) initial perceptions toward mosquito host-seeking traps. Results indicate participants use household-level strategies for insect control that reveal familiarity with the concept of spatial repellent and contact irritant actions of chemicals and that placing traps in the peridomestic environment to remove repelled mosquitoes was acceptable. Preliminary evidence suggests a Push-Pull strategy should be well accepted in these locations. These results will be beneficial for developing future large scale push-pull interventions and are currently being used to guide insecticide application strategies in (entomological) proof-of-concept studies using experimental huts.

## Introduction

Dengue viruses cause more human morbidity and mortality worldwide than any other arthropod-borne virus, and represent the most rapidly advancing vector-borne disease in the world.^1^–^3^ Infections produce a spectrum of clinical illness ranging from a nonspecific viral syndrome to severe and fatal hemorrhagic disease. Annually, there are an estimated 50–100 million cases of dengue fever (DF) worldwide, up to 500,000 cases of dengue hemorrhagic fever (DHF), and over 24,000 deaths (mainly among children) attributed to dengue viruses.^1^,^4^

The four different dengue serotypes are maintained in a cycle that involves humans and *Aedes* mosquitoes (principally *Aedes aegypti*). The most common strategies for dengue vector control worldwide focus on reducing vector populations through larviciding and/or container removal. However, the needs to seek out, identify, and treat or remove all larval development sites make the implementation of these strategies challenging. Indoor residual or space spray techniques, such as thermal fogging and ultra-low volume (ULV) spraying, are also used for controlling adult *Ae. aegypti*.^5^–^7^ These measures are implemented at the time or shortly after an epidemic has been identified to serve as emergency control but, although these may have a dramatic effect in reducing the numbers of reported dengue cases for a transient time following application, are not used for disease prevention.^8^ In addition to the logistical complexities local vector control authorities have in implementing these strategies (i.e., financial and labor constraints, infrastructure limitations, and public willingness to allow access to enter homes), insecticide resistance within the vector population can warrant a once effective killing agent ineffective thereby decreasing the available chemical tools recommended for vector control. However, evidence exists that resistant populations exhibit repellent and irritant behavioral responses to insecticides independent of their toxic effects.^9^ Combined, these indications warrant the evaluation and development of novel vector control strategies. These new approaches should ideally include the integration of currently available insecticides, based on other chemical actions they possess, into appealing consumer-based products for increased cost-benefit. This includes the use of insecticide-treated materials that can be applied at the household level.^2^,^10^,^11^

This study represents one component of a larger proof-of-principle research program designed to evaluate the effectiveness of reducing indoor densities of *Ae. aegypti* using minimal treatment coverage and dose of spatial repellents and contact irritant chemicals currently recommended for vector control—specifically, chemical tools registered for public health either through indoor residual spray (IRS) or insecticide-treated bed net (ITN) interventions, such as alphacypermethrin, deltamethrin, lambda-cyhalothrin, permethrin, and DDT (reference compound).^12^,^13^ These chemicals are being evaluated at doses and coverage levels that exploit spatial repellent (SR) and contact irritant (CI) actions with minimal toxicity to reduce insecticide resistance selection pressure—such an approach deviates from current adult control strategies, which focus on a direct chemical kill. Applied at the house level, an SR action will prevent mosquitoes from entering a home, and a CI will promote mosquito escape from indoors. Both behaviors will reduce indoor *Ae. aegypti*, reducing human-vector contact, and thereby potentially prevent dengue transmission. As this approach will allow the adult mosquito to move freely within the outdoor environment, we are exploring the use of an outdoor trap to augment an SR or CI strategy by subsequently removing repelled/irritated vectors from the peridomestic environment (i.e., the development of a “Push-Pull” system). We have chosen to evaluate the BG-Sentinel trap (BGS; Biogents AG, Regensburg, Germany), as the “Pull” component, because of previously reported efficacy in capturing *Ae. aegypti* under field settings compared with standard human landing collections and other reference trapping devices (i.e., Fay-Prince).^14^–^16^ Two study sites have been chosen to evaluate the Push-Pull strategy: Iquitos, Peru and Kanchanaburi, Thailand.

A novel aspect of this strategy is the inclusion of the intent to repel mosquitoes without a direct chemical killing action. One possible barrier to this strategy is that householders potentially might perceive the intervention as ineffective, and adoption of the strategy might be compromised. Thus, regardless of how efficacious such a control strategy may prove to be during proof-of-concept, it is critical to assess the potential barriers and/or acceptance levels of local populations living in the area where implementation trials may occur during post-project development. No strategy will be successful, regardless of how effective, if the affected population does not believe in it and adopt it

In preparation for scale up following proof-of-concept, focus groups were conducted in both Peru and Thailand as an initial assessment to acceptability. We explored baseline perceptions toward the Push-Pull system, including specific components of this system (i.e., repellent/irritant-treated material, trapping devices, etc.), acceptable options for product delivery (i.e., treated textile, plastics, sprays, coils, oils, etc.), attitudes toward mosquitoes in the domestic environment, and current mosquito control practices and expenditures on control products. This is the first known assessment of perceptions regarding a spatial repellent and/or contact irritant vector control strategy designed for implementation at the house level.

## Methods

### Ethics statement.

Institutional Review Board (IRB) approval for this study was obtained from the Uniformed Services University of the Health Sciences (USUHS) for both locations. In addition, the following IRB approvals were obtained for Peru: U.S. Naval Medical Research Center Detachment in Lima (NMRCD) and Tulane University School of Public Health and Tropical Medicine. Approvals for Thailand came from the Ethical Committee for Research in Human Subjects, Department of Disease Control, Ministry of Public Health.

### Study settings.

This study took place in two dengue endemic communities: Iquitos, Peru and Lad Yaa, Thailand.

#### Iquitos, Peru.

**Figure 1.**
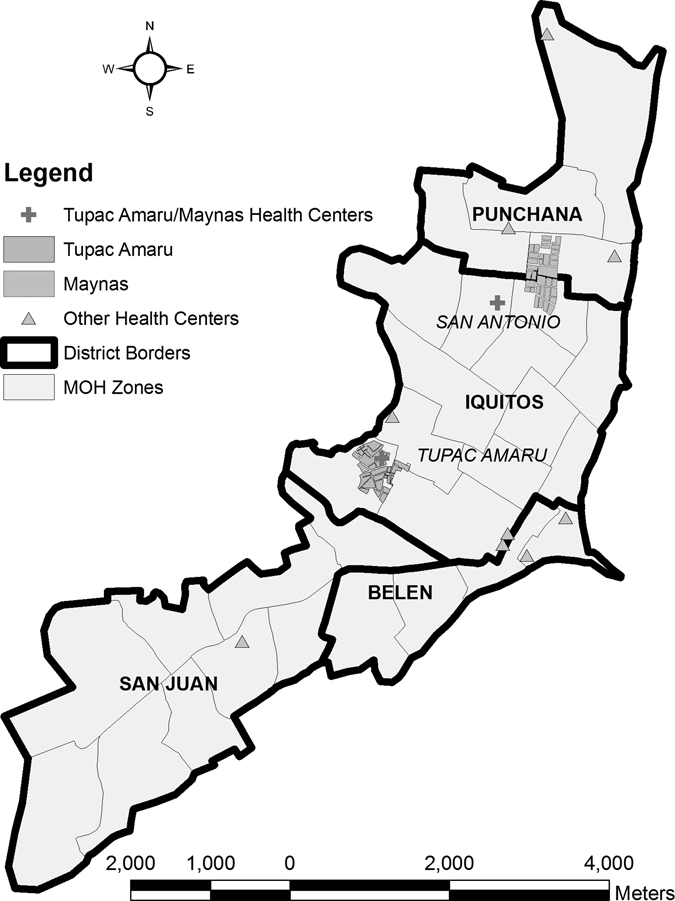
Iquitos, Peru study location from which inhabitants of the two neighborhoods, Maynas and Tupac Amaru, were recruited for participation in focus group discussions.

Situated in the northeastern Loreto Department of Peru, Iquitos has a population of 370,000 distributed throughout four main districts: San Juan, Iquitos, Punchana, and Belen (Figure 1).^17^ Focus group participants were recruited from two Iquitos neighborhoods, Maynas and Tupac Amaru, which have been described in detail previously.^18^–^20^ Each is served primarily by the San Antonio and Tupac Amaru Health Centers, respectively, where NMRCD has been conducting febrile surveillance studies since 2000 (Figure 1 and Table 1). Both sites experience dengue burden with Maynas typically having higher *Ae. aegypti* population densities and dengue transmission rates than Tupac Amaru: the 1999 age adjusted seroprevalence rates for dengue were 89% and 69%, respectively.^20^–^23^ In 2007, seroprevalence rates to dengue-3 (predominant serotype circulating from 2002 to 2007) were 47% in Maynas and 37% in Tupac Amaru (ACM, personal communication). Both neighborhoods show wide variability in housing conditions and since 2002, have been the first areas to report cases during dengue outbreaks. These neighborhoods were selected because they are being monitored as part of ongoing longitudinal studies and thus there was established contact in these neighborhoods and existing household census data; residents are monitored for both febrile illness (3 times per week), serologically (6-month intervals), and entomologically (household *Ae. aegypti* surveys every 4 months).

#### Lad Yaa, Thailand.

**Figure 2.**
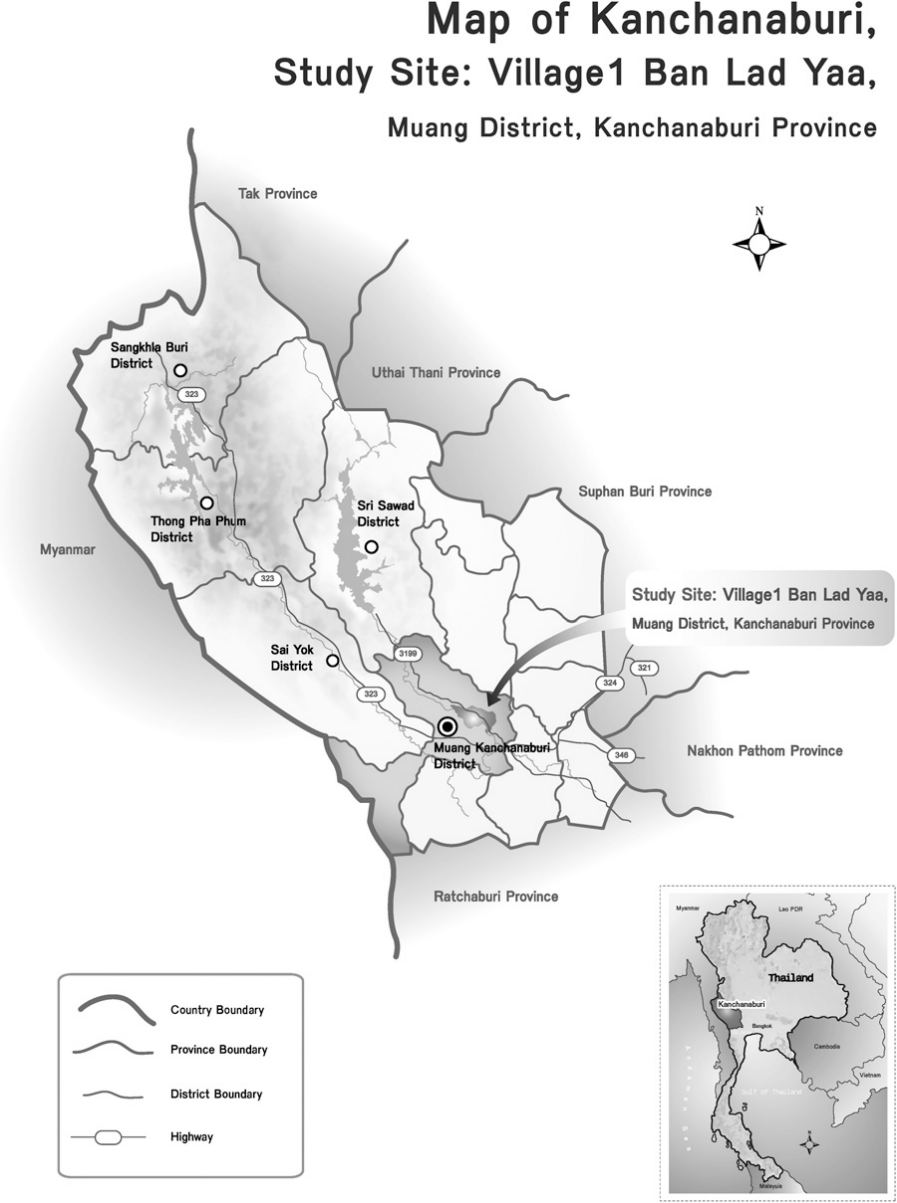
Kanchanaburi, Thailand study location from which inhabitants of Ban Lad Yaa within the Muang District were recruited for participation in focus group discussions.

Lad Yaa is situated in the Muang District of Kanchanaburi Province in west-central Thailand (Figure 2). Approximately 129 km (80 miles) from Bangkok, Kanchanaburi is the third largest province of Thailand and shares a 370-km-long border with Myanmar on the west. The province is divided into 13 districts, 95 cantons, and 959 villages. The majority (95%) of the population draw their income from agriculture including citrus, rice, and rubber. The average income is estimated at THB73,231 (US$2,154) per annum, ranking 31st countrywide. Muang district was chosen as the study site because of continuous dengue endemicity in the last 6 years: 2003–2008.^24^ Study volunteers were recruited from Lad Yaa canton of Muang district as the canton has the highest dengue incidence of the district (Table 1). Residents of Lad Yaa engage mainly in small to medium business, or work for the government, with a small proportion working in agriculture.^24^ The canton can be described as semi-urban, comprised mostly of permanent housing structure, shop houses, or stand-alone houses. Like all of the semi-urban settings in Thailand, residents of Lad Yaa have ready access to government-operated health facilities.

### Sampling criteria.

Purposive sampling was used to select focus group participants in both locations: all participants were adult permanent residents of locations where experimental hut studies are being performed for entomological evaluation of the Push-Pull strategy, and held decision-making power within the household (i.e., not an employee of the house). Participants were recruited on the basis of age and gender to ensure representation of opinions and practices of these different groups, and focus group discussions were stratified by age groups and gender to increase probability of volunteer participation (i.e., facilitate ease of expressing opinions).

### Recruitment approach.

The recruitment process varied between the two study locales on the basis of previous recruiting experiences in these areas and existing rapport with the respective community.

#### Iquitos, Peru.

Because of previous experience with low volunteer turnout when recruitment occurs days in advance, research team members traveled to study communities for recruitment ~2 hours before a particular scheduled focus group. Persons meeting the age and gender inclusion criteria were recruited at their homes within a randomly selected 10 contiguous block area of the two different neighborhoods. In this house-to-house recruitment, if an individual met the inclusion criteria and was available for participation for a specific focus group, research assistants then explained study goals using the approved recruitment script. The goal was to have ~10 participants per focus group, thus recruitment stopped when the assistants had met the quota. Recruited participants were then instructed to meet at a central location within their communities for transportation to the NMRCD Iquitos field laboratory. Informed consent was conducted at the NMRCD laboratory before the start of each focus group discussion.

#### Lad Yaa, Thailand.

One week before the interview dates, the research assistants visited every fifth house on the house registration list obtained from the local health center and determined whether the adult family member present met the age and gender inclusion criteria; if so, the trained assistants explained the goal of the study to those individuals, and the recruitment script was left with them. Up to 12 individuals were invited per focus group, knowing some would elect not to participate. On the day of a particular focus group, participants who agreed to volunteer were transported to a central location for informed consent before the focus group discussions.

### Samples.

In all, 102 individuals, between 25 and 65 years of age, participated in nine focus group discussions (5 groups in Peru and 4 groups in Thailand) (Table 2). The variation in total focus groups performed between the study sites reflects the use of saturation, defined as the point at which no new themes or information emerges during the data collection that was used to guide sampling in both sites.^25^ Saturation is a commonly used threshold for sample size in qualitative research.^25^

### Methodology.

Each social scientist convened a local research team for the focus groups within each country, composed of a note taker and an assistant. The principal investigator (PI) participated in focus group discussions being facilitated by the social scientists to ensure that any additional information that emerged during the discussions that might be useful to the project could be explored in more depth at that time. Each session started with the facilitator (the site-specific Lead Social Scientist) describing the purpose of the larger research program and focus group study in either fluent Spanish or Thai, accordingly. This was followed by the IRB approved site-specific informed consent process: oral consent in Peru and written consent in Thailand. Facilitators presented graphics that displayed: 1) the Push-Pull strategy concept; 2) the experimental hut design used for conducting the entomological evaluations; and 3) the peridomestic trap (i.e., BG-Sentinel trap). The facilitators described the study, allowed for questions, and then led discussions using a focus group guide to ensure that the same topics were discussed in all groups. The focus group guide was developed by the PI (NA, a medical entomologist) in conjunction with the Peruvian and Thai Lead Social Scientists (VPS and VP) based on five main themes of interest: 1) attitude toward mosquitoes in the domestic environment; 2) current mosquito control practices; 3) current expenditures on mosquito control products; 4) perceptions of spatial repellency and contact irritancy versus killing mosquitoes; and 5) initial perceptions toward mosquito host-seeking traps placed in the peridomestic environment. The facilitators also displayed varying types of textiles that are currently being used in entomological evaluations to quantify mosquito behavior against chemical treatments. The local research assistants took detailed notes and audiotapes were used to record each discussion. Upon completion of each focus group, the study team met to discuss participant outcome statements within the structure of the five pre-established key themes and supplement handwritten notes with audio recordings (to make sure all topics of discussion were noted and to add relevant quotes).

### Data management and analysis.

Detailed reports for all focus groups were organized on the basis of the main themes of the study as outlined in the focus group guide: 1) attitude toward mosquitoes in the domestic area; 2) household mosquito control practices; 3) current expenditures for mosquito control products; 4) acceptability of spatial repellency and contact irritancy versus killing mosquitoes; and 5) initial perceptions toward mosquito host-seeking traps placed in the peridomestic environment. Each research team met to document all issues discussed regarding each theme, then the site-specific lead social scientist summarized these trends.^26^,^27^ Quotes that were representative of key ideas expressed are presented here to depict the language and ideas of the participants, in their own words.

## Results

### Attitude toward mosquitoes in the domestic area.

Mosquitoes were described as a problem in all focus groups conducted in both Peru and Thailand. Participants were aware of the health risk associated with mosquito contact and stated that, beyond being simply a nuisance, mosquitoes in and around their homes could transmit dengue and other arthropod-borne diseases, such as malaria and filariasis.

The concern about dengue from participants in Iquitos was universal: all expressed concern about acquiring infection, and many mentioned that a person in their home and/or someone that they knew had had dengue. Most men and women also discussed that mosquitoes are most often found in the lower parts of walls, in dark areas or rooms of one's home, such as under beds and/or in closets, on doors and windows and framed pictures, and in humid locations, such as bathrooms and kitchens. Women did not discuss feeling the presence of mosquitoes in their yards or in the back of their houses, whereas the younger group of men (25–40) discussed the presence of many mosquitoes outdoors, in the bushes and trees of their backyards.

Findings from Thailand were similar to those from Peru, in that all study participants reported feeling uneasy about mosquitoes in the domestic and peridomestic areas of their homes because of nuisance biting, discomfort, and potential serious health risks. The participants also demonstrated good understanding of locations within their houses where mosquitoes are located: dark and damp corners in rooms, in closets, and especially in bathrooms. Because they are aware of the health risks posed by mosquitoes, all participants in Thailand were keen on protecting their family members from being bitten. A representative quote from a young mother illustrates this point: *“I am very careful with my child. I am afraid that she will get sick with dengue… I would do anything to prevent my child from being bitten by mosquitoes. I spend a lot of money buying things to ensure that my child is safe from mosquito bites.”*

### Household mosquito control practices.

Study participants from both sites clearly demonstrated routine mosquito control practices that were associated with cost. Adult control was managed using various techniques including commercially available mosquito coils, plug-in emanators, and indoor space spray—although these methods for adult mosquito control were used less frequently than petroleum in Iquitos (see next paragraph) and electric fans in Lad Yaa. Environmental management was also described for larval control. Canned aerosol insecticides appeared to be commonly used in both locales. Although insecticide spray was considered to work well, participants reported feeling uneasy about using these products because of perceived human toxic effects, and all agreed that they would welcome alternative methods. In Iquitos, both men and women expressed concerns regarding human toxicity (especially exposure for children): *“I know [it is toxic] because of the strong smell. If we [adults] can smell it, it is only worse for children.”* However, one woman stated her family had started using insecticide spray because of reports of an DHF case in their neighborhood, which scared them, so it was worth the risk and the cost.

Like their Peruvian counterparts, Thai heads of households with young children and elderly reported concerns regarding toxicity from canned aerosol insecticides: *“I use a lot of aerosol insecticides that I bought from the store to kill indoors mosquitoes. It's necessary because otherwise there will be a lot of mosquitoes in my house. Mosquitoes are bad because they bite and can also bring diseases to us. So, even if insecticide is not good, I am more afraid of getting dengue than insecticide toxicity. I just make sure that kids and elderly are not near where I use insecticide.”*

A common household strategy unique to the Iquitos study site was the use of petroleum or creoline, applied to cement floors using a mop or sprinkled on the ground (if the floor is made of dirt), both indoors and outdoors, including front sidewalks, to keep mosquitoes away. This was the most popular practice by far in all groups, and even for those that did not use petroleum, everyone was familiar with this practice. One man described: *“…it [petroleum] keeps the mosquitoes away because it has a strong smell – it doesn't kill them, just keeps them away.”* Petroleum was used daily in most homes in different combinations—alone, with bleach or detergent, and sometimes mixed with wax. Those using it with wax specified that though it was more expensive (because of the cost of wax), the effects lasted longer: both in keeping the shine on the cement floor and in keeping mosquitoes away. In addition to its application to the floors, participants also used petroleum in places where mosquitoes are regularly seen resting: the lower sides of walls, behind framed pictures, and on doors, door frames, and windows.

In contrast to Peru, participants in Thailand used electric fans to reduce mosquitoes from landing on them. Because of year-round warm weather, electric fans are a common household item, and are used to provide comfort indoors. Other adult mosquito control methods described in Thailand included burning of organic material (dried citronella, citrus fruit zest, leaves from the garden, etc.), and application of topical repellent gels and creams—although restricted to more affluent families.

Overall, there was general consensus among study participants from both study sites that the government-run indoor space spray programs do not work. The most common explanation by Peruvian participants for the failure of these campaigns was that the insecticide mixture was “too dilute” or that they sprayed too infrequently. A few felt the indoor space spray program was effective for a few days but the effects do not last long. Several people felt the indoor space sprays only “angered” the mosquitoes, and that biting became more aggressive after homes were sprayed. In Thailand, fogging is implemented once a year just before the rainy season and, like their Peruvian counterparts, the participants detest the method, and reported that the method does not work: *“It's very loud… It smells bad, too… Worse yet, the effects are short-lived, sometimes only one day, and then mosquitoes come back. I don't like it [fogging].”*

### Current expenditures on mosquito control products.

Participants in both study sites are currently purchasing household mosquito control products. Moreover, when asked hypothetically about their willingness to pay for a Push-Pull strategy as described to them, most in both sites stated being willing to do so.

Participants in Iquitos mentioned that although the canned aerosol insecticides reduce mosquitoes within their domestic environment, the cost (about US$3–$4 per can, which lasts a week) is prohibitive. Those who simply sprinkle petroleum or creoline on a daily basis on their floors spend about US$0.30/day. There were a few women who mentioned they mixed it with wax because it “stays longer” (both the shine and the smell), therefore did not have to be applied as frequently, but this also added about US$1 to the cost, which most were unwilling to spend. However, when asked if they would be willing to spend on a product that would last longer but would require a more significant investment, most said they would. Participants stated there are certain household items that are not in their day-to-day budget, but for which they must save to make the purchase. They explained that if a product were to be made available that works, but for which they would need to pay once every certain number of months versus daily, that this would be acceptable. One woman in her 30's explained: *“For most of our household expenses, we pay a little bit daily, but for others, we know we have to set some money aside for things that will cost more, but that we only need to buy occasionally. We are used to having to save a little for that. For example, if you have a gas stove, every so often, you have to buy a new tank of propane gas [for cooking], and though it is expensive, you only do it once in a while.”*

Participants in the Thailand site reported being willing to pay a minimum of THB1,060 (US$31) and maximum of THB2,500 (US$73) to ensure protection from mosquito bites. Participants reported using several cans of commercially available aerosol insecticide per year, ranging from one can every 2 months to two cans every month (6–24 cans per year). All the Lad Yaa study participants knew of the use of topical repellent creams and agreed that the products are effective, but the cost was stated as prohibitive for those with less disposable income. Only households with small children or elderly and more affluent would use this strategy. One young mother with a toddler reported buying repellent cream from Japan. She explained: *“The living area in my house is not enclosed, so mosquitoes are everywhere. I am very concerned that my child would come down with dengue… Yes, it's [repellent cream] costing me dearly, but I would do anything for my baby.”*

### Spatial repellency and contact irritancy versus killing mosquitoes.

As the participants in the focus groups in both study sites began to talk, it was clear that they already apply the concept of spatial repellency and contact irritancy in their current household mosquito control strategies. Volunteers in all focus groups from Iquitos knew that the petroleum or creoline primarily used on their floors did not kill mosquitoes, but simply kept them away. In addition, using the petroleum or creoline in their homes was not limited to the floor: women specifically talked about applying these products in key places in their homes to target mosquitoes. These sites included doors, door frames, windows, and window frames because the mosquitoes were observed to rest on those spots, and treating these areas will prevent them from entering and/or resting indoors. When asked to discuss further about whether they needed to see evidence of a dead mosquito to feel that a product worked, it was clear that as long as they did not feel or see the presence of mosquitoes in the places the insecticide was applied, they would feel the product worked.

Another interesting example that the concept of spatial repellency is common in Iquitos came up unexpectedly, on a topic unrelated to mosquitoes. As the focus groups talked about types of products that they hang or place in their homes that could be adapted to contain or be treated with a spatial repellent product, people mentioned the *“water bag used to keep flies away.”* When probed further, participants mentioned that it is a common practice in homes, and especially in places that serve food, to place a clear plastic bag filled with water either above or on tables to keep flies away. When the bag becomes dusty, it is replaced with a new bag. When asked to describe how this tool actually worked to prevent flies from approaching the area, the common response was that *“it scares the flies away.”* In fact, the use of water-filled bags to keep pest insects out of a specified area has also been described anecdotally in other tropical countries.

As for the Thailand participants, the use of electric fans were widely used and accepted to keep mosquitoes away from an indoor space, but not kill them: *“Whenever and wherever I sit down in my house, I turn on the fan. It makes me feel comfortable as it cools me down, and it also blows the mosquitoes away. On certain days I could have up to four electric fans around me to make sure that mosquitoes cannot come near me (saying with laughter).”* In addition, the burning of organic materials or mosquito coils was conducted outdoors at the threshold of the domestic area to prevent mosquitoes from entering and the majority of the participants reported having screened bedrooms to reduce mosquito populations inside the rooms.

### Response to a peridomestic trap.

Three main issues regarding a trap in the outdoor area were of concern in all focus groups from both study sites: color, safety, and placement. Cost of the trap, particularly if an attractant lure is needed, was a concern specifically of participants from Thailand. Participants both in Peru and Thailand stated that traps should be made of dark colors—brown or black—simply because dark colors attract mosquitoes. Safety concerns focused on the possibility that young children may come into contact with moving parts or lure components. Older participants (40–65 years) concluded that placing the trap inside a protective cage, such as chicken coops, which are already available locally, would make the trap accessible to mosquitoes but not children, and that this cage could be sold as an accessory to the trap. However, there was concern that additional work needed to properly set up the trap in the home environment would reduce the likelihood of a person to buy the product: the easier to use, the better.

Regarding placement of the trap, the focus was to keep it out of contact with animals and/or children. Various suggestions were offered, such as hanging it from high places (trees, beams of houses, etc.) or placing it on a high wall, but some participants stated that this may defeat the purpose of the trap because mosquitoes do not rest in high places. Other discussions about trap placement related to maintenance and upkeep. Participants stated protection of trap components would be important to reduce exposure to heavy rainfall, which is typical in both locales. They suggested placing traps in a location not exposed to precipitation (i.e., under ledges, etc.), or making available trap covers as a separate accessory. One woman suggested using palm leaves to cover the cages holding the traps, because mosquitoes are attracted to the palm leaves.

To enhance acceptability, suggestions were made to place the traps in public places that are frequented, such as plazas and parks, to increase the familiarity of people with the trap and potentially motivate people to use them in their home environment. Thai participants specifically suggested providing a brief explanation next to the trap to reduce public suspicion. Participants suggested getting the municipal authorities to purchase the traps for homeowners to ensure standardization of implementation and facilitate acquisition (i.e., concerns that the trap and lure would be cost-prohibitive). Interestingly, one male participant from Thailand reported the making and using of a homemade mosquito trap, he explained: *“I have a fruit orchard and I make my own traps for pests in my garden all the time. Making a mosquito trap is not much more difficult. I just use a large tin can and paint the inside black and put some dirty (used) clothes with lots of sweat in the can as lure, and leave it all day and night in a dark and damp corner. In the morning or whenever I am free I'll go and execute the trapped mosquitoes by placing the “mosquito electric racket” over the can and lightly tap the can so that mosquitoes would fly out and therefore are electrocuted.”* This account impressed all members in his group and they became excited about the idea of trapping mosquitoes, especially when one member mentioned that he heard that dead mosquitoes can be sold to feed fish at about THB700 (US$20) per kilogram.

## Discussion

Despite efforts from community based and/or vertically structured *Ae. aegypti* control programs, the global trend in the incidence of dengue fever has steadily continued to increase over the past decade.^28^ These trends indicate that evaluation and development of novel vector control strategies, targeting both the larval and adult mosquito populations, are needed. Because of limited financial and personnel resources available for most vector control programs, such strategies ideally should include the integration of currently available public health tools into appealing consumer-based products for increased cost-benefit. This includes the use of insecticide-treated materials that can be applied at the household level.^2^,^10^,^11^

The objective of these focus group discussions was to gather qualitative baseline data regarding the initial acceptability of a novel Push-Pull strategy for *Ae. aegypti* control in two dengue-endemic locales: Iquitos, Peru and Kanchanaburi, Thailand. The goal of this study was not to compare participant answers between the two sites but rather to characterize a wide range of attitudes toward the proposed Push-Pull strategy. Similarities between locations were highlighted solely to show that the approach may have acceptance within two distinct cultures: the two sites where proof-of-principle of the larger research program will take place. Although Thai participants were recruited a week before the actual focus group discussion, they were not informed of the topics that would be discussed in advance, which may have biased results. The beliefs, practices, and opinions expressed by participants in both locales were based on life-long daily experiences.

It was evident in the current study that participants from both study sites were very familiar with mosquito behavior, based both on comments made when discussing mosquitoes in their communities, and based on the strategies they had adopted to control mosquitoes in their houses. For instance, in Iquitos, Peru, the petroleum and/or creoline products were applied along door and window frames to prevent mosquitoes from entering through these portals and along the floorboards to reduce the likelihood of mosquito resting on these surfaces. Although there is no entomological evidence that these work, people perceive that these repel mosquitoes and have identified key locations to keep them out of their homes. In Thailand, participants explained that they focus attention on dark corners when implementing household vector control strategies. The sites for application of control tools were selected as a result of observational evidence of mosquito presence by homeowners. This finding is supportive of the likely community acceptance of an intervention that focuses on placing an insecticide treated product around portals of entry or preferred resting sites (i.e., “Push” components): such an intervention would resonate with their current observations and knowledge of mosquito behavior and, in fact, reflects the study design approach currently being used in our experimental hut evaluations.

The current adult *Ae. aegypti* control in Iquitos, Peru and Kanchanaburi, Thailand involves thermal fogging, ULV and/or indoor residual spray techniques to serve as emergency, rather than preventive, measures.^5^–^7^ Although these programs may be effective in reducing mosquito populations, the general attitude of study participants from both sites was negative toward government-run vector control operations, specifically for those that require entry into households for application (i.e., ULV indoor sprays, thermal fogging). These perceptions should serve as an indicator that mosquito control strategies designed for implementation by the local homeowner may prove highly acceptable in these locales. If the homeowner does not perceive government-run schemes to be effective, they may rely on other means to reduce mosquitoes within the house. This includes purchasing and using consumer-based products (i.e., household level interventions), which could include components of the Push-Pull strategy. Perhaps more importantly, providing effective, adaptable, and accessible vector control tools, in addition to government-run programs, will promote both partnership and transfer of ownership of health protection to homeowners and may have an additive benefit in reducing disease. Future research planned in these sites should explore people's perceptions regarding whose responsibility it is to control mosquitoes: whether it should be each household or the government or a related institution. The authors recognize the ability to control pest or nuisance mosquitoes is an important component to integrated vector control programs because of end-user acceptability and sustainability issues—and people may perceive this Push-Pull strategy is failing if they observe other nuisance mosquitoes in their homes. The current program is using *Ae. aegypti* as the model system for proof-of-concept, however, if successful, a Push-Pull strategy is expected to have an impact on other mosquito genera and/or species but this will have to be evaluated in post-project experiments.

It was clear in our focus groups that all households spent money on products to control mosquitoes in the domestic area and the information on their current expenditures for these products can help guide the development of the form of delivery by defining the limits an individual resident is willing to invest in mosquito control. Currently, the households in the Peru study locale spend ~US$100/year on products, but purchase them on a daily basis. In the Thailand study site, participants reported spending slightly less, ranging from US$31.1 and $73.5 per year. Because the overall Push-Pull strategy being evaluated incorporates residual chemicals with characteristics of long-term efficacy, it is expected that people would need to invest in a product that would be purchased once every 3 to 6 months with an estimated daily expenditure similar to what they spend now. Although an estimate of the Push-Pull strategy cost can only be defined once the optimum form of delivery is identified, this one-time purchase would require larger lump sums of money spent at a time, which may be a problem for some. Though people in both study locales expressed willingness-to-pay for such a product, there are several limitations associated with this particular question that warrant discussion. First, we were asking about a hypothetical mosquito control system and it may be hard for people to really assess willingness-to-pay unless they can see or experience what they are being asked to purchase. Moreover, though participants reported that they would be willing to spend more on a product that lasted longer, it is unclear if this is truly the case because many participants in Peru did not use the petroleum and wax combination partly because of its perceived higher expense—despite the fact that because it does not need to be used on a daily basis it actually adds up to about the same cost as the non-wax combination. And third, participants might have overstated their willingness-to-pay simply to please the research team, and/or to not “appear poor” among fellow focus group participants.

It became apparent from the focus groups that it will be important to identify an optimum form of delivery that can be adapted by the end user either for potential secondary uses (i.e., a product that makes the home look neater or cleaner or smell nicer) or by how the end product unit is sold (i.e., by length of rope, one poster, etc.) to provide people with different purchasing options. The primary reason participants in Peru applied petroleum mixtures inside homes was for mosquito control but secondary benefits included making the floor shine (if cement), controlling dust (if unpaved), and improving home odor (with the addition of fragrance or bleach etc.). Similar findings about the importance of secondary benefits of mosquito control products in their sustained use have been reported from bed net studies in the Peruvian Amazon where results indicate bed nets were used for other reasons beyond protection from mosquitoes, including warmth, privacy, and a sense of security for children.^29^ During the focus groups, we explored ideas for forms/methods of delivery of the “Push” component, which would be acceptable in those particular communities. For example, in Peru, we started with the originally envisioned delivery option: an insecticide-treated textile that could be hung near doors and windows and on the lower parts of walls. Because we initially presented some form of “textiles” as a delivery option, some groups started talking about household items of the same material hanging in their houses—curtains, cloth calendars, etc. But then some groups discussed further and commented on disadvantages of textiles (e.g., acquisition of dirt). Other groups talked about entirely different delivery platforms such as insecticidal paints. Although the cloth displayed during focus groups was chemical-free and effectiveness of such an intervention had not started, the research team wanted to ensure that the Push-Pull *concept* would be considered an acceptable strategy in the targeted communities to identify specific challenges to program success. In sum, the feedback from the focus groups has provided new ideas about potential forms of delivery “interfaces” (format in which the “Push” component of strategy would be delivered) that we can use to explore, develop, and test in current entomological evaluation studies.

The level of support shown by study participants may be due in part to hearing of a “new” product, such that any new strategy would have been well received, reflecting the distress mosquitoes are exerting on these populations. In addition, because the PI was present during focus group discussions participants may have been compelled to show enthusiasm for the strategy. However, it is rare for a local population to be consulted for their opinions about how a vector control strategy might be applied, and volunteers exhibited pleasure in having been provided an opportunity to voice their opinions and express their concerns. More than once, when the focus group discussion ended, people volunteered to take the research team to their community, so that we would meet and talk to others, and view the living conditions in their neighborhoods to understand the challenges in strategy implementation under specific living conditions (i.e., house structure design). Some participants expressed their desire to test the strategy at their home during post-project development because if it worked, they told us, they would experience first-hand how their ideas had helped create a novel product designed to increase public health and would be the first to promote it to others. In other words, in addition to identifying conceptual acceptance and perspectives, the focus group discussions generated a sense of ownership in strategy development.

Qualitative data from the current study indicate that mosquito control strategies that involve spatial repellency and contact irritancy are not only acceptable among the populations enrolled, but are actually used by participants in both settings. This suggests persons in locations where the Push-Pull strategy will be tested following proof-of-concept may find the idea of repelling an insect (rather than requiring direct kill of mosquitoes) quite “normal.” Whether the current local practices are actually effective is irrelevant: on a daily basis, people invest time and money in practices that they perceive controls the mosquitoes in their homes, and these practices include chemical actions other than toxicity. People's perception of the effectiveness of a strategy is often associated with adoption of a protective behavior. For example, other studies on use of impregnated bed nets for malaria control, one of which was conducted in Peru, Ecuador, and Colombia, revealed that use of the bed nets was associated with people's perception that it would protect them from disease.^30^ The overwhelmingly positive response to the Push-Pull strategy in this initial assessment suggests that strategies designed to reduce vector-borne diseases using non-killing actions of chemicals could be accepted in these communities. That said, this study outlines an initial assessment to acceptability within a limited participant population: we plan to examine acceptability in these communities by survey methods once there is an actual intervention that people are expected to place and maintain in their homes. These surveys will assess factors associated to adoption and maintenance behaviors of this strategy, and identify barriers to its correct and consistent use, to ensure (or improve) its sustainability.

In conclusion, the current study was not designed or intended to measure risk assessment by local populations regarding mosquito control methodologies, or to assess whether these beliefs and practices are consistent or logical. If the Push-Pull strategy proves to be effective in reducing indoor densities of adult *Ae. aegypti* mosquitoes, then in-home pilot trials will be conducted to refine the optimum form of delivery and production schemes. However, the integration of a social science assessment into the larger medical entomology research program at the front end of the project has provided baseline insight into the acceptability of the Push-Pull concept and viable options for delivery on the basis of cultural traditions and preferences (i.e., mosquito control practices, housing structure and beautification, etc.) that a strictly entomological research program would have missed. These results will be integrated into current proof-of-concept studies in experimental huts by exploring and evaluating discussed delivery options for the “Push” component of the strategy, taking into consideration concerns and discussions associated with the “Pull” component, and beginning to identify specific challenges in post-project implementation at each study site. This information is most beneficial in the early stages of experimentation to optimize the experimental approach in evaluating the product/concept during the course of the study, and creating a sense of ownership in the communities for potential upcoming products.

## Figures and Tables

**Table 1:** Description of study settings outlining key characteristics related to vector control and disease transmission

City, country	Study site	Population as of 2007	Dengue cases	Economic status	Housing type	Main types of occupation
Iquitos, Peru	Maynas	14,657	174*	Slightly higher than Tupac Amaru	Range from simple wood structures (30%) with thatch roof, to concrete block or brick with corrugated metal roofs	Mixed commercial, agriculture, and professional
	Tupac Amaru (TA)	11,032	146*		Range from simple wood structures (40%) with thatch roof, to concrete block or brick with corrugated metal roofs	Mixed commercial, agriculture, and professional
Kanchanaburi, Thailand	Lad Yaa	30,161	101†	THB60,000‡ (US$1,764)	Mostly permanent, shop houses, with some stand-alone houses	Predominately agriculture, mixed small business, local government positions

* Cases captured in 2006–2008.

† Confirmed cases, Jan–Dec 2008 (of this dengue fever (DF) = 61, dengue hemorrhagic fever (DHF) = 36, and dengue shock syndrome (DSS) = 4); Source: Provincial Health Office, Kanchanaburi.

‡ Average annual income; Source: Lad Yaa Local Administrative Organization, 2009.

**Table 2:** Demographic characteristics of focus group participants (age and gender) by study site

		Male		Female			Total
Study city and country	Study site	25–40	41–65	25–40	25–50	41–65
Iquitos, Peru	Maynas	–	10	15	–	–	**25**
	Tupac Amaru	12	–	–	12	13	**37**
Kanchanaburi, Thailand	Lad Yaa	9	10	10	–	11	**40**
**Total**		**21**	**20**	**25**	**12**	**24**	**102**
